# Reliability of preoperative MRI findings in patients with lumbar spinal stenosis

**DOI:** 10.1186/s12891-021-04949-4

**Published:** 2022-01-15

**Authors:** Hasan Banitalebi, Ansgar Espeland, Masoud Anvar, Erland Hermansen, Christian Hellum, Jens Ivar Brox, Tor Åge Myklebust, Kari Indrekvam, Helena Brisby, Clemens Weber, Jørn Aaen, Ivar Magne Austevoll, Oliver Grundnes, Anne Negård

**Affiliations:** 1 Department of Diagnostic Imaging, Akershus University Hospital, Lørenskog, Norway; 2 Institute of Clinical Medicine, University of Oslo, Oslo, Norway; 3 Department of Radiology, Haukeland University Hospital, Bergen, Norway; 4 Department of Clinical Medicine, University of Bergen, Bergen, Norway; 5 Unilabs Radiology, Oslo, Norway; 6 Hofseth BioCare, Ålesund, Norway; 7 Department of Orthopaedic Surgery, Ålesund Hospital, Møre and Romsdal Hospital Trust, Ålesund, Norway; 8 Division of Orthopaedic Surgery, Oslo University Hospital Ulleval, Oslo, Norway; 9 Department of Physical Medicine and Rehabilitation, Oslo University Hospital, Oslo, Norway; 10 Department of Research and Innovation, Møre and Romsdal Hospital Trust, Ålesund, Norway; 11 Department of Registration, Cancer Registry of Norway, Oslo, Norway; 12 Kysthospitalet in Hagevik. Orthopaedic Clinic, Haukeland University Hospital, Bergen, Norway; 13 Department of Orthopaedics, Sahlgrenska University Hospital, Gothenburg, Sweden; 14 Department of Orthopaedics, Institute for clinical sciences, Sahlgrenska Academy, University of Gothenburg, Gothenburg, Sweden; 15 Department of Neurosurgery, Stavanger University Hospital, Stavanger, Norway; 16 Department of Quality and Health Technology, University of Stavanger, Stavanger, Norway; 17 Department of Circulation and Medical Imaging, Faculty of medicine and health sciences, Norwegian University of Science and Technology, Trondheim, Norway; 18 Department of Orthopaedics, Akershus University Hospital, Lørenskog, Norway

**Keywords:** Reliability, Lumbar spinal stenosis, Interobserver agreement, Intraobserver agreement, MRI

## Abstract

**Background:**

Magnetic Resonance Imaging (MRI) is an important tool in preoperative evaluation of patients with lumbar spinal stenosis (LSS). Reported reliability of various MRI findings in LSS varies from fair to excellent. There are inconsistencies in the evaluated parameters and the methodology of the studies. The purpose of this study was to evaluate the reliability of the preoperative MRI findings in patients with LSS between musculoskeletal radiologists and orthopaedic spine surgeons, using established evaluation methods and imaging data from a prospective trial.

**Methods:**

Consecutive lumbar MRI examinations of candidates for surgical treatment of LSS from the Norwegian Spinal Stenosis and Degenerative Spondylolisthesis (NORDSTEN) study were independently evaluated by two musculoskeletal radiologists and two orthopaedic spine surgeons. The observers had a range of experience between six and 13 years and rated five categorical parameters (foraminal and central canal stenosis, facet joint osteoarthritis, redundant nerve roots and intraspinal synovial cysts) and one continuous parameter (dural sac cross-sectional area). All parameters were re-rated after 6 weeks by all the observers. Inter- and intraobserver agreement was assessed by Gwet’s agreement coefficient (AC1) for categorical parameters and Intraclass Correlation Coefficient (ICC) for the dural sac cross-sectional area.

**Results:**

MRI examinations of 102 patients (mean age 66 ± 8 years, 53 men) were evaluated. The overall interobserver agreement was substantial or almost perfect for all categorical parameters (AC1 range 0.67 to 0.98), except for facet joint osteoarthritis, where the agreement was moderate (AC1 0.39). For the dural sac cross-sectional area, the overall interobserver agreement was good or excellent (ICC range 0.86 to 0.96). The intraobserver agreement was substantial or almost perfect/ excellent for all parameters (AC1 range 0.63 to 1.0 and ICC range 0.93 to 1.0).

**Conclusions:**

There is high inter- and intraobserver agreement between radiologists and spine surgeons for preoperative MRI findings of LSS. However, the interobserver agreement is not optimal for evaluation of facet joint osteoarthritis.

**Trial registration:**

www.ClinicalTrials.gov identifier: NCT02007083, registered December 2013.

**Supplementary Information:**

The online version contains supplementary material available at 10.1186/s12891-021-04949-4.

## Background

Symptomatic lumbar spinal stenosis (LSS) is the leading cause of spine surgery in individuals over 65 years of age [1]. The condition is caused by narrowing of the lumbar spinal canal, the lateral recesses and/or the neural foramina, often due to degenerated facet joints and intervertebral discs or thickened flaval ligaments [2]. The diagnostic accuracy of MRI and its association with clinical symptoms and surgical outcome of patients with LSS is controversial [3–5]. Nevertheless, MRI helps to select patients for surgery [6], confirm the diagnosis and assess the severity of LSS, define the anatomic location of the stenosis (e.g. central, lateral recess or foraminal), and rule out other conditions that may mimic symptoms of LSS [7]. By localising the apparent cause of the stenosis (e.g. a bulging disc or thickened flaval ligaments), the various MRI findings of LSS can influence the surgical strategy [8]. Therefore, reliable interpretation of MRI findings is an important step towards offering appropriate treatment to patients and to provide optimal information for the surgeon; a shared understanding of the MRI findings between surgeons and radiologists facilitates information exchange and treatment decisions. Previous studies have reported the reliability of different features of MRI in LSS. However, the reported reliability estimates vary widely, even for the same parameters [9–12]. While some studies have included fair number study subjects, other studies vary greatly in the number and the medical specialty of the observers who performed the studies [9, 13–16]. In a systematic review, Andreisek et al. [17] found reported observer agreement values for facet joint osteoarthritis varying from poor to excellent; reliability data were lacking for hypertrophy of the flaval ligaments, redundant nerve roots (RNR) of the cauda equina and reduction of the posterior epidural fat. This lack of consensus on the reliability of MRI findings and insufficient reliability data for some parameters highlights the need for studies with higher quality, higher number of the included patients, and observers from relevant medical specialties.

The current study was based on prospective imaging of patients scheduled for surgical treatment of LSS. We hypothesised that given agreement upon the definitions of the MRI parameters used in the diagnosis of LSS, the observer reliability would be good. Thus, the aim of this study was to evaluate the reliability of the commonly used, preoperative MRI findings in the assessment of foraminal and central canal stenosis, facet joint osteoarthritis, RNR and intraspinal synovial cysts, using observers with different levels of experiences.

## Methods

The participants in this cross-sectional study were consecutively included from the Spinal Stenosis Trial of the NORwegian Degenerative spondylolisthesis and Spinal STENosis (NORDSTEN) study, a prospective, multicentre, randomised controlled trial that was designed to compare different surgical treatments for LSS [18]. Patients with clinical and radiological findings consistent with LSS were referred to an orthopaedic or neurosurgical outpatient clinic. In total, 437 patients are included in this trial. The inclusion and exclusion criteria are presented in Table 1. All patients were provided written informed consent before inclusion. The current study was performed in accordance with the declaration of Helsinki and adhered to the ICMJE recommendations for the protection of research participants. The study adheres also to the *Guidelines for Reporting Reliability and Agreement Studies (GRAAS)* [19].

**Table 1:** Inclusion and exclusion criteria

Inclusion criteria	Exclusion criteria
Age between 18 and 80 years, clinical symptoms of LSS, not responding to at least 3 months of non-surgical treatment, radiological findings (foraminal, central canal or lateral recess stenosis) corresponding to the clinical symptoms such as back pain, leg pain or neurologic claudication, and understanding the Norwegian language (spoken and written).	Previous surgery at the level of stenosis, previous fracture or fusion of the thoraco-lumbar spine, cauda equina syndrome (bowel or bladder dysfunction) or fixed complete motor deficit, ASA (American Society of Anesthesiologists) grade 4 or 5, more than 20° lumbosacral scoliosis, distinct symptoms in lower limbs due to other diseases, stenosis in more than three lumbar levels, being unable to comply fully with the protocol, isthmic defect in pars interarticularis at the level of stenosis, participation in another clinical study that could interfere with the present trial, alcohol or substance abuse and ≥ 3 mm spondylolisthesis verified on upright lateral view X-ray.

### Sample size

The “kappa size package” of the R statistics software (Version 1.2, 2009–2019 RStudio Inc. Boston, USA) was used to calculate the required sample size. Assuming a prevalence of 0.4 and 0.6 in each of the categories of a dichotomous outcome parameter, a power of 80% and a significance level of 5%, a sample size of 102 is required to estimate a kappa (κ) of 0.8 with a 95% Confidence Interval (CI) of 0.7–0.9 for agreement between four independent observers. To account for possible losses, we enrolled 108 consecutive patients in this study.

### Imaging

Imaging was performed between February 2013 and March 2015. Since the NORDSTEN study is a large multicentre study involving 18 hospitals, MRI examinations were performed in 1.5 or 3 Tesla units from different manufacturers. To maintain a certain level of quality of the examinations, the performing institutions were provided standard MRI protocols including sagittal T1- and axial and sagittal T2- weighted images. The MRI sequences were performed with repetition time / echo time: 400–826 / 8–14 ms for T1-weighted images and 1500–6548 / 82–126 ms for T2-weighted images, slice thickness: 3–5 mm, field of view: 160–350 mm. All radiological examinations were anonymised, without any link to the demographic or clinical information, the imaging institution, or the manufacturer of the MRI unit.

### Image evaluation

MRI examinations were evaluated by two orthopaedic spine surgeons (observers 1 and 2, with ten and six years of experience, respectively) and two radiologists (observers 3 and 4 with 13 and 12 years of experience in musculoskeletal imaging, respectively). They evaluated the images independently and re-evaluated all the images in a random order after a minimum of 6 weeks. This time interval was chosen to assure independency of the test-retest reads. Each observer assessed the image quality for every parameter. If an image was evaluated as inadequate for one or several parameters by one or several observers, that parameter was excluded for all observers. In this way, only parameters with test and retest results from all four observers were included in the final analysis.

The observers used integrated measurement tools in a Picture Archiving and Communication System (IDS7 PACS, Sectra, Sweden) for all measurements. All observers measured the angle between the axial images and a line passing through the centre of the intervertebral disc at each level on the sagittal images. The axial images with more than five degrees of angulation with the intervertebral discs were excluded from the statistical calculations. Proposed classification systems for foraminal stenosis (by Lee et al. [20]), central canal stenosis (by Schizas et al. [21]), and facet joint osteoarthritis (by Weishaupt et al. [22]) were used. Presence or absence of intraspinal synovial cysts [7] and RNR [23] were also rated. To improve the clinical applicability, we dichotomised these parameters into two categories: 0 (normal or mild pathology) and 1 (moderate or severe pathology). The methods used for the categorical ratings and the frequency distribution of these findings are presented in Table 2. Additionally, the observers measured the dural sac cross-sectional area (DSCA) quantitatively at the level of the intervertebral disc. The measurement methods used in this study are presented in the additional file (Grading and measurement methods).

**Table 2:** Descriptions and frequency distributions of the categorical parameters

Parameter	Severity of degenerative changes	Examined parameters by all observers (%)^**a**^
**Foraminal stenosis according to Lee et al.** **[**20**]**	**Category 0** (Lee grade 0 and 1): no obliteration of perineural fat, or obliteration only horizontally or vertically	2019 (88)
	**Category 1** (Lee grade 2 and 3): obliteration of perineural fat both horizontally and vertically, with or without structural changes in the nerve	277 (12)
**Central canal stenosis according to Schizas et al.** **[**21**]**	**Category 0** (Schizas grade A and B): inhomogeneous or grey fluid signal, recognisable rootlets, posterior epidural fat is present	605 (63)
	**Category 1** (Schizas grade C and D): grey or black signal from the central canal, no recognisable rootlets, posterior epidural fat may or may not be present	363 (37)
**Facet joint osteoarthritis according to** **Weishaupt et al.** **[**22**]**	**Category 0** (Weishaupt grade 0 and 1): normal, or mild joint space reduction and hypertrophy of the articular process, small osteophytes	681 (34)
	**Category 1:** (Weishaupt grade 2 and 3): moderate or marked joint space reduction and hypertrophy of the articular process with osteophytes, erosions or cysts	1335 (66)
**Redundant nerve roots (RNR)**	**Category 0**: normal appearance of the cauda equina	941 (79)
	**Category 1**: tortuous nerves of the cauda equina	243 (21)
**Intraspinal cysts**	**Category 0**: no synovial cysts	1959 (99)
	**Category 1**: presence of intraspinal synovial cyst	25 (1)

^a^Counts and percentages for each parameter as separated by horizontal lines. For foraminal stenosis, facet joint osteoarthritis and intraspinal cysts left and right sides are accounted

In a pilot study of ten randomly selected patients from the study population, all observers rated all the categorical parameters and measured the DSCA. The rating criteria and procedures were discussed in two joint meetings between all observers, one before and one after the pilot study. The measurements from the pilot study were not included in the statistical calculations.

### Statistical analyses

For the categorical parameters, distributions across the observers were assessed. Gwet’s agreement coefficient (AC1) [24] with 95% CIs was used for computing the inter- and intraobserver agreement. This coefficient is preferred instead of κ when the measurements are not normally distributed, which was the case here (to avoid the so-called high agreement low κ paradox) [25]. For the DSCA, the Intraclass Correlation Coefficient (ICC) with 95% CIs was used to calculate the inter- and intraobserver agreement and the Bland-Altman analysis to estimate the mean differences with 95% limits of agreement.

We used STATA software (StataCorp. 2017. Stata Statistical Software: Release 15. College Station, TX: StataCorp LLC) and its user-written package *kappaetc* for the statistical calculations. Data from different levels and sides from the same patient were treated as independent observations in the analyses. Distribution of data was assessed by visual inspection of the plots.

### Interpretation of the agreement values

AC1 values were interpreted using a scale for κ defined by Landis and Koch [26], as proposed by Gwet [27], to indicate poor (≤ 0.0), slight (0.01–0.20), fair (0.21–0.40), moderate (0.41–0.60), substantial (0.61–0.80) or almost perfect (0.81–1.00) agreement. ICC values were interpreted to indicate poor (< 0.50), moderate (0.51–0.75), good (0.76–90) and excellent agreement (> 91) [28].

## Results

**Fig. 1:**
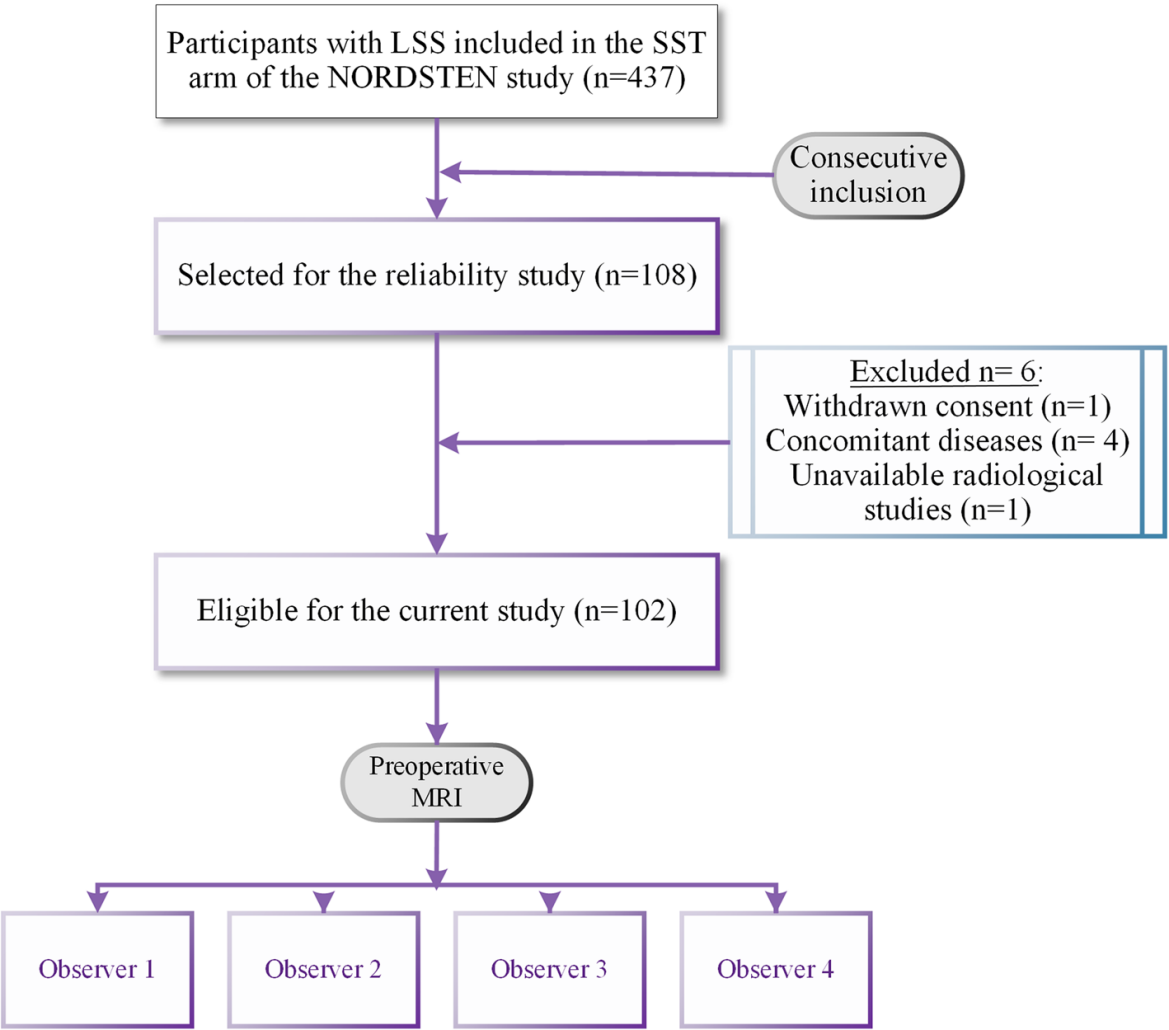
The flow chart of the study demonstrates the inclusion process for the study and causes of the exclusions. LSS: Lumbar Spinal Stenosis, SST: Spinal Stenosis Trial

In this study, six of the 108 (6%) consecutively enrolled participants were excluded. A flow chart demonstrating the inclusion process and the causes of the exclusions is presented in Fig. 1.

**Fig. 2:**
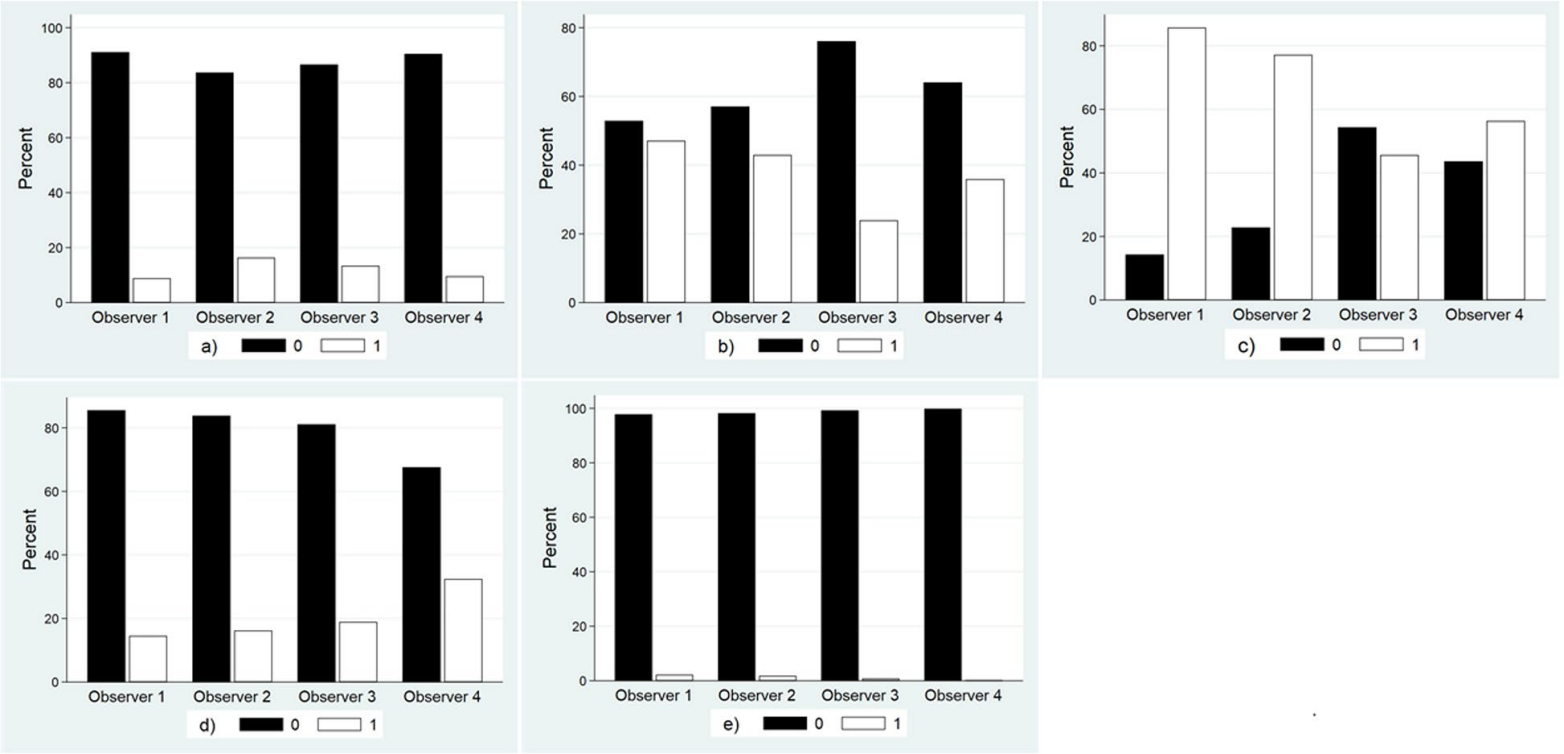
Frequency distribution of the categorical parameters: **a** foraminal stenosis according to Lee et al. **b** central canal morphology according to Schizas et al. **c** facet joint osteoarthritis according to Weishaupt et al. **d** redundancy of the cauda equina and **e** intraspinal synovial cysts. The values for a, b and c are dichotomised. Category 0 indicates absent or mild pathology and category 1 indicates moderate or severe pathology

The total number of the rated parameters (after excluding those parameters without ratings from all observers) is presented in Table 2. The mean age ± standard deviation for the study participants was 66 ± 8 years (66 ± 8 for men and 66 ± 9 for women) and 53 (52%) were men. Bar graphs demonstrating the frequency distribution of the categorical ratings across observers are presented in Fig. 2.

**Fig. 3:**
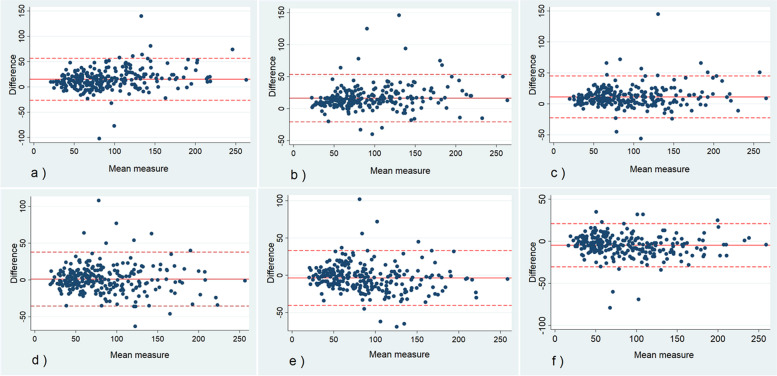
Bland-Altman plots demonstrating the degree of agreement and variability of the measurements of the dural sac cross-sectional area (DSCA) between observers 1 and 2 (**a**), 1 and 3 (**b**), 1 and 4 (**c**), 2 and 3 (**d**), 2 and 4 (**e**) and 3 and 4 (**f**). The solid horizontal lines show the mean differences, and the dashed lines show 95% limits of agreement

Using the Bland-Altman method, the mean value of the DSCA was calculated for each of the six observer pairs (from the first ratings) and plotted against the corresponding differences (Fig. 3).

### Interobserver agreement

The overall interobserver agreement was substantial or almost perfect for all categorical parameters (AC1 0.67–0.98, 95% CI range 0.60 to 0.97), except for facet joint osteoarthritis. While the surgeons demonstrated substantial agreement for facet joint osteoarthritis (AC1 0.72), the agreement for the other observer pairs and the overall agreement was fair to moderate (AC1 0.22–0.48). Both pairwise and overall agreements for the DSCA were good or excellent (ICC 0.86–0.96, 95% CI range 0.53–0.97). The DSCA measurements (for both stenotic and non-stenotic levels) ranged from 13 to 283 mm^2^ and the pooled mean differences between the observers was 5.9 mm^2^ (95% limits of agreement ±35.5 mm^2^). The overall and pairwise results of the interobserver agreement are summarised in Table 3.

**Table 3:** Interobserver agreement

Parameter	Observer 1 and 2	Observer 1 and 3	Observer 1 and 4	Observer 2 and 3	Observer 2 and 4	Observer 3 and 4	All observers
**Lee grade**	0.82 (0.78–0.86)	0.88 (0.85–0.91)	0.90 (0.87–0.93)	0.78 (0.74–0.83)	0.82 (0.78–0.86)	0.86 (0.83–0.90)	0.85 (0.82–0.87)
**Schizas grade**	0.74 (0.65–0.82)	0.53 (0.42–0.64)	0.72 (0.63–0.80)	0.61 (0.51–0.71)	0.75 (0.67–0.84)	0.71 (0.62–0.80)	0.67 (0.60–0.74)
**Weishaupt grade**	0.72 (0.66–0.78)	0.23 (0.13–0.32)	0.39 (0.31–0.48)	0.22 (0.13–0.31)	0.48 (0.40–0.56)	0.30 (0.22–0.39)	0.39 (0.33–0.45)
**Redundant nerve roots (RNR)**	0.82 (0.76–0.88)	0.84 (0.78–0.89)	0.68 (0.60–0.76)	0.77 (0.70–0.84)	0.64 (0.55–0.73)	0.68 (0.60–0.76)	0.74 (0.69–0.80)
**Intraspinal cysts**	0.97 (0.95–0.98)	0.98 (0.96–0.99)	0.98 (0.97–0.99)	0.98 (0.97–0.99)	0.98 (0.97–0.99)	0.99 (0.99–1.0)	0.98 (0.97–0.99)
**DSCA**	0.86 (0.64–0.93)	0.88 (0.53–0.95)	0.91 (0.77–0.95)	0.91 (0.90–0.93)	0.92 (0.90–0.94)	0.96 (0.93–0.97)	0.91 (0.86–0.94)

Gwet’s agreement coefficient (AC1) is used for calculation of the interobserver agreement for the categorical parameters and Intraclass Correlation Coefficient (ICC) for the DSCA (Dural Sac Cross-sectional Area). 95% confidence intervals are given in the parentheses

### Intraobserver agreement

The intraobserver agreement was substantial or almost perfect for all the categorical parameters (AC1 0.63–1.00, 95% CI range 0.56 to 1.0) and excellent for the DSCA (ICC 0.93–1.00, 95% CI range 0.91 to 1.0) for all observers (Table 4). The mean intraobserver difference for the DSCA measurements was 0.17, 95% limits of agreement agreement were ± 34.7.

**Table 4:** Intraobserver agreement

Parameter	Observer 1	Observer 2	Observer 3	Observer 4
**Lee grade**	0.97 (0.96–0.99)	0.84 (0.81–0.88)	0.91 (0.88–0.93)	0.95 (0.93–0.97)
**Schizas grade**	0.86 (0.80–0.93)	0.80 (0.73–0.88)	0.78 (0.70–0.85)	0.98 (0.96–1.00)
**Weishaupt grade**	0.91 (0.89–0.94)	0.72 (0.66–0.78)	0.63 (0.56–0.70)	0.97 (0.95–0.99)
**Redundant nerve roots (RNR)**	0.95 (0.91–0.98)	0.78 (0.72–0.85)	0.90 (0.86–0.94)	0.95 (0.92–0.99)
**Intraspinal cysts**	0.99 (0.98–1.00)	0.97 (0.95–0.98)	0.99 (0.99–1.00)	1.00 (1.00–1.00)
**DSCA**	0.98 (0.98–0.99)	0.93 (0.91–0.94)	0.96 (0.95–0.97)	1.00 (1.00–1.00)

Gwet’s agreement coefficient (AC1) is used for calculating the intraobserver agreement for the categorical parameters and Intraclass Correlation Coefficient (ICC) for the DSCA (Dural Sac Cross-sectional Area). 95% confidence intervals are given in the parentheses

## Discussion

In this study, two musculoskeletal radiologists and two orthopaedic spine surgeons with different levels of experience assessed a set of preoperative MRI parameters that may influence the surgical strategy of patients with symptomatic LSS. We found high levels of inter- and intraobserver agreement for all of parameters but for facet joint osteoarthritis. Although previous studies have examined the reliability of various MRI findings in patients with LSS, the reported results vary from low to high for some parameters [9–12]. In the current study, we included a large number of patients and four observers with different levels of experiences. To enhance the quality of the study, we used suggested GRAAS guidelines for reporting reliability studies [19].

We used a classification system proposed by Weishaupt et al. [22] for evaluation of facet joint osteoarthritis, but achieved only moderate overall interobserver agreement, comparable to the results reported by Weishaupt et al. In a study of 100 patients with symptomatic LSS, Winklhofer et al. [29] evaluated ten qualitative and quantitative parameters on MRI, using two radiologists as observers. They found moderate to substantial interobserver agreement for qualitative parameters describing central canal and foraminal stenosis or nerve root impingement (κ 0.42–0.77) and good agreement for measurement of the DSCA (ICC 0.85). For a dichotomous grading of facet joint osteoarthritis (yes /no), the interobserver agreement was only fair (κ 0.27) and the intraobserver agreement was good (κ = 0.69). The authors speculated that this low interobserver agreement might be related to the challenges of differentiating mild from no osteoarthritis on MRI. In a study by Carrino et al. [11] of 111 patients with lumbar radiculopathy who were candidates for surgery, the interobserver agreement between three experienced radiologists and one spine surgeon was comparable to our results for osteoarthritis of the facet joints (κ = 0.54); the intraobserver agreement was good (κ = 0.69). The authors in the study by Carrino et al. used a four-point consensus-based grading system (normal, mild, moderate, and severe) on MRI. To our knowledge, high reliability values for grading the severity of osteoarthritis of the facet joints on MRI has not been reported. This inferior reliability is worth to note and discuss. Our experience is that degenerative changes in the facet joints cause elongation of the joint spaces in different directions and evaluating these changes on a single axial slice of MRI is challenging. Using multiple axial images (or using them in combination with sagittal images) may improve this evaluation. Previous research indicates slightly more favourable reliability for evaluation of facet joint osteoarthritis on Computed Tomography (CT) than on MRI [30] and we suggest using CT whenever this modality is available. Similar to the studies of Winklhofer et al. and Carrino et al., the results of the current study are limited to patients with symptomatic LSS. We included therefore only MRI parameters that directly or indirectly could influence the surgery, making our results relevant to preoperative findings in patients with symptomatic LSS. In line with a previous report [31], we found good/ excellent inter- and intraobserver agreement for intraspinal synovial cysts. When mimicking symptoms of LSS by causing radiculopathy, intraspinal synovial cysts are treated surgically [7] and a preoperative MRI is necessary to evaluate the status of the adjacent facet joint and the nerve root. Our reliability estimates for presence of RNR were similar to those reported by Papavero et al. [32] for their four-part classification of cauda equina redundancy. This finding is believed to associate with the level(s) of central canal stenosis [33]. In the current study, observer 1 and 4 (a surgeon and a radiologist) demonstrated very high intraobserver agreement values (0.96 to 1.0), but the interobserver values between these observers for Schizas score, PNR and particularly for Weishaupt score were not high. This may indicate different understanding of these findings on MRI and thus an inferior validity of these measurement methods. Observer four demonstrated very high intraobserver agreement for several parameters including the Weishaupt score. This observer is a highly experienced musculoskeletal radiologists who has experience form orthopaedic surgery as well. This wide range of experience may explain the high agreement values.

High observer agreement between experienced radiologists and spine surgeons is important to identify the most relevant MRI findings and to offer appropriate treatment to patients with LSS, yet few reliability studies have considered observers from both specialities to obtain comparable reliability data. Low reliability of the imaging findings may contribute to the existing differences in surgical decision making for these patients [34, 35]. The generally good overall agreement between experienced radiologists and spine surgeons in our study is reassuring for clinical work and research. For example, our results suggest that the differences in DSCA measurements between the observers were < 6 mm^2^ on average and < 42 mm^2^ in 95% of cases, when evaluating both stenotic and non-stenotic levels. This limited observer variability is relevant in clinical practice because it indicates that experienced radiologists and spine surgeons perform comparable DSCA measurements.

In a systematic review, Andreisek et al. [17] identified 14 radiological parameters used by researchers for evaluating LSS. This review revealed a wide range of reported κ values (0.01 to 1.00) for inter- and intraobserver agreement. Further, the authors pointed out that the definitions of current categorical imaging criteria for LSS were vague. To address this issue, we used the classification systems of Lee et al. [20], Schizas et al. [21] and Weishaupt et al. [22]. Several of the 14 parameters identified by Andreisek et al. can be recognised in the classification systems we used. For example, the authors reported lack of reliability data for hypertrophy of the flaval ligaments and reduction of the epidural fat, both recognisable through the Schizas classification that we used and demonstrated good reliability for.

In contrast to our results, Speciale et al. [9] demonstrated poor to moderate inter- and intraobserver agreement for evaluation of central canal and foraminal stenosis of patients with symptomatic LSS between seven observers with wide range of experiences. The authors argued that the lack of agreement upon the definitions of these parameters at the outset of the study was the cause of this inferior reliability. It is unclear whether joint meetings between the observers in our study was a cause of higher agreement. However, agreement on nomenclature used, instructional courses for less experienced physicians and maintenance discussions in multidisciplinary meetings between radiologists and spine surgeons may contribute to better observer agreement. In our opinion, the results of the present study and previous studies highlight the need for general radiologists and other health professionals who evaluate MRI examinations of patients with LSS to be updated on the nomenclature.

### Limitations

The patients included in the current study were diagnosed with LSS and scheduled for surgery. This knowledge may have introduced bias for the observers during the image evaluations. However, this is the typical clinical scenario for preoperative evaluation of patients with LSS. Selecting patients from a randomised controlled trial with specific inclusion and exclusion criteria may limit the external validity of the results. On the other hand, this was a conscious choice made by our group to increase the internal validity of the results and to address earlier studies reporting low reliability of radiological findings in LSS [9, 10]. Inclusion of patient from a randomised trial may also have introduced some selection bias in the examined sample. However, the performed evaluations in this study included normal, as well as abnormal levels.

## Conclusions

The inter- and intraobserver reliability of preoperative MRI findings including stenosis parameters, RNR and intraspinal synovial cysts in patients with LSS is generally good. However, we found only fair or moderate interobserver agreement for facet joint osteoarthritis.

## Supplementary Information

**Additional file 1.** Grading and measurement methods. Explanation of the grading and measurement methods used in the curret study.

## Data Availability

Due to restrictions from the Norwegian Data Inspectorate, the complete dataset cannot be published. However, data produced during the current study may be assessed through the corresponding author upon a reasonable request.

## Abbreviations

AC1: Gwet’s Agreement Coefficient
CI: Confidence Interval
CT: Computed Tomography
DSCA: Dural Sac Cross-sectional Area
ICC: Intraclass Correlation Coefficient
LSS: Lumbar Spinal Stenosis
κ: Kappa
MRI: Magnetic Resonance Imaging
NORDSTEN: NORwegian Degenerative spondylolisthesis and spinal STENosis
RNR: Redundant Nerve Roots
SST: Spinal Stenosis Trial
