# Supplementary material for: Reliability of preoperative MRI findings in patients with lumbar spinal stenosis
Source: BMC Musculoskelet Disord. 2022 Jan 15;23:51. doi: 10.1186/s12891-021-04949-4 (PMC8760672; doi:10.1186/s12891-021-04949-4)
Supplement: Supplementary file 1 — Additional file 1. Grading and measurement methods. Explanation of the grading and measurement methods used in the curret study. [file 12891_2021_4949_MOESM1_ESM.docx]

**Reliability of preoperative MRI findings in patients with lumbar spinal stenosis**

Grading and measurement methods used.


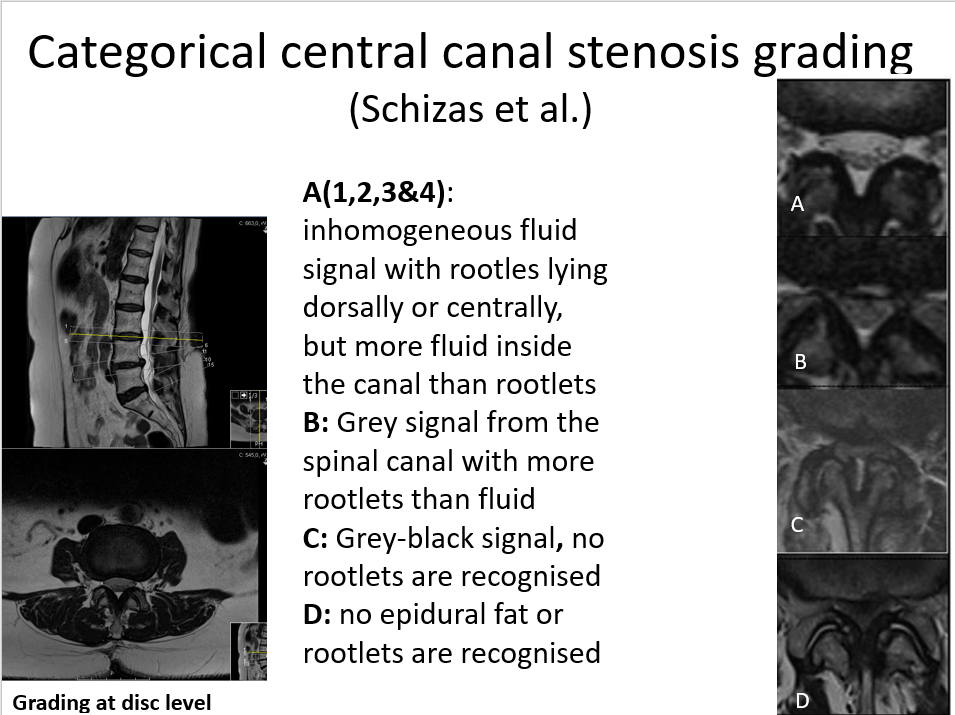


*Schizas et al. (2010) Qualitative grading of severity of lumbar spinal stenosis based on the morphology of the dural sac on magnetic resonance images. Spine (Phila Pa 1976)*


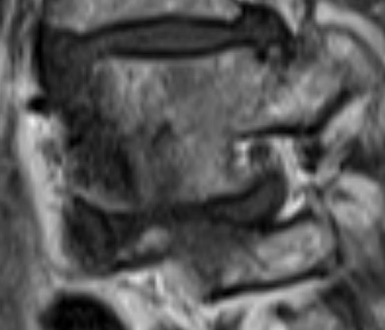

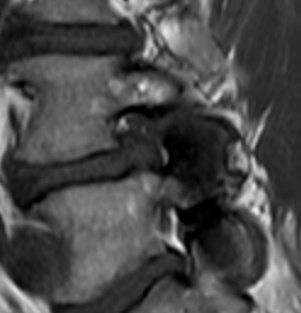

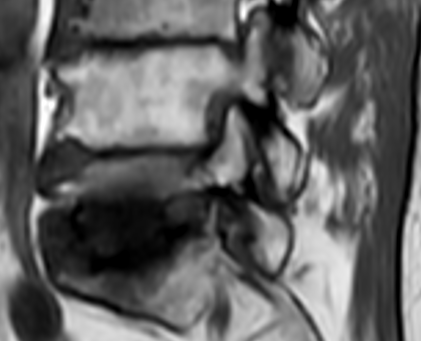

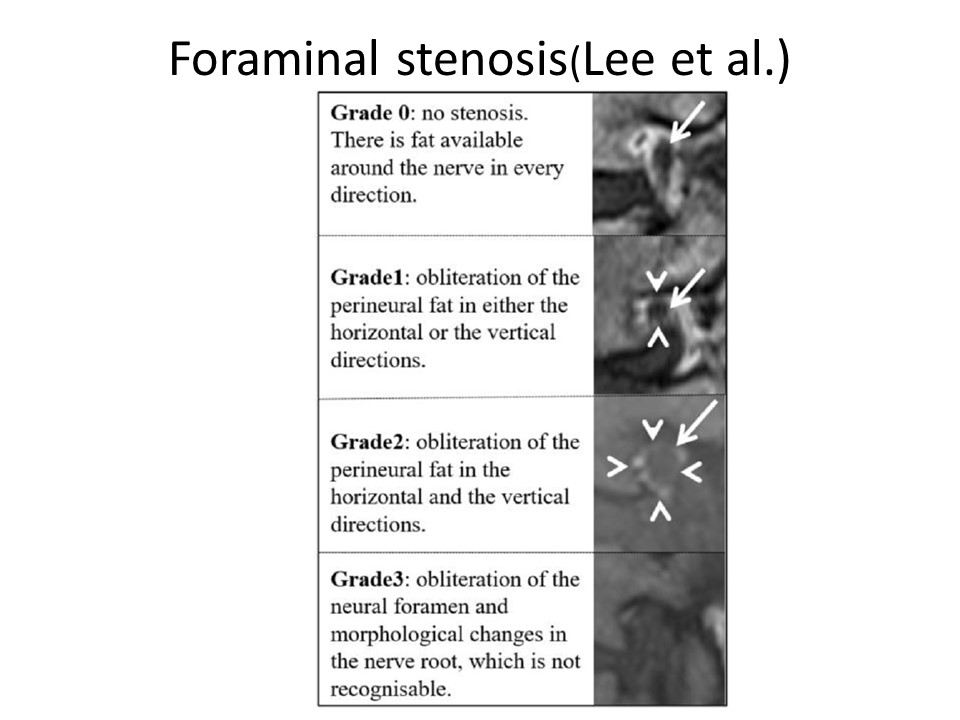


*Lee et al. (2010) A practical MRI grading system for lumbar foraminal stenosis. AJR Am J Roentgenol 194:1095–1098.*


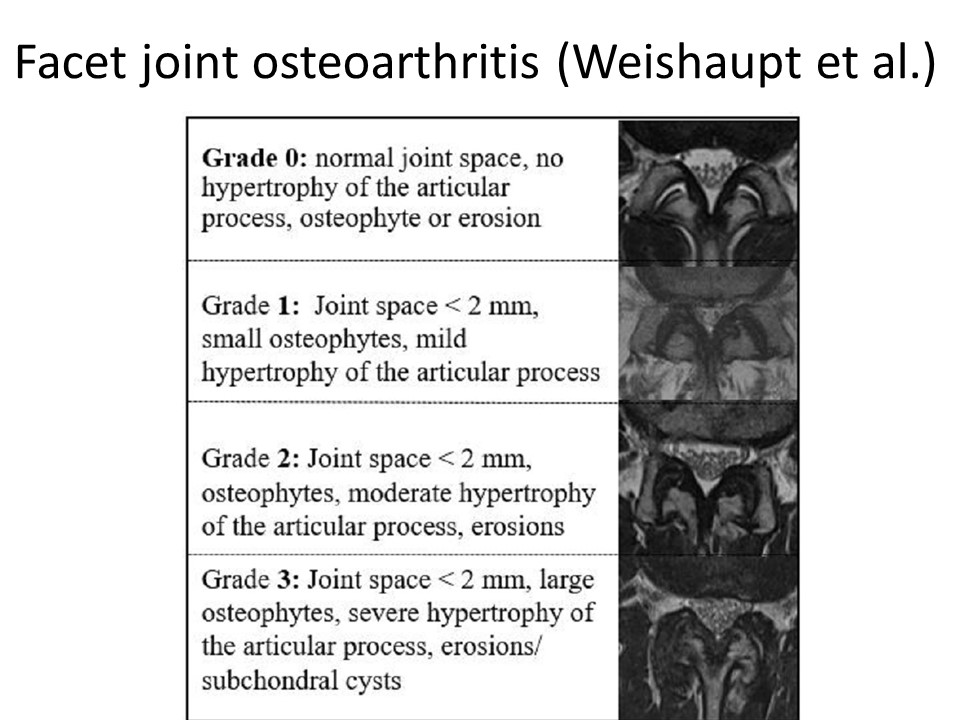


*Weishaupt et al. (1999) MR imaging and CT in osteoarthritis of the lumbar facet joints. Skeletal Radiol 28:215–9*


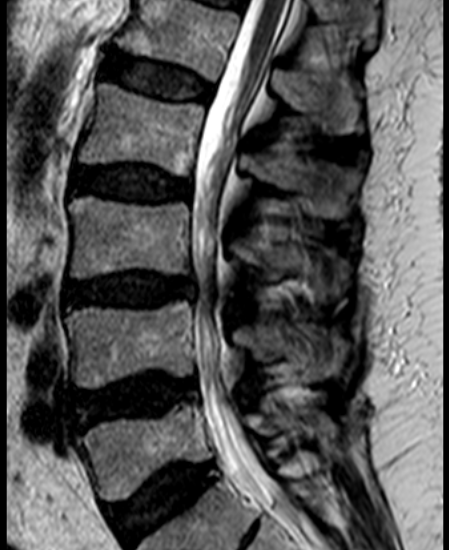

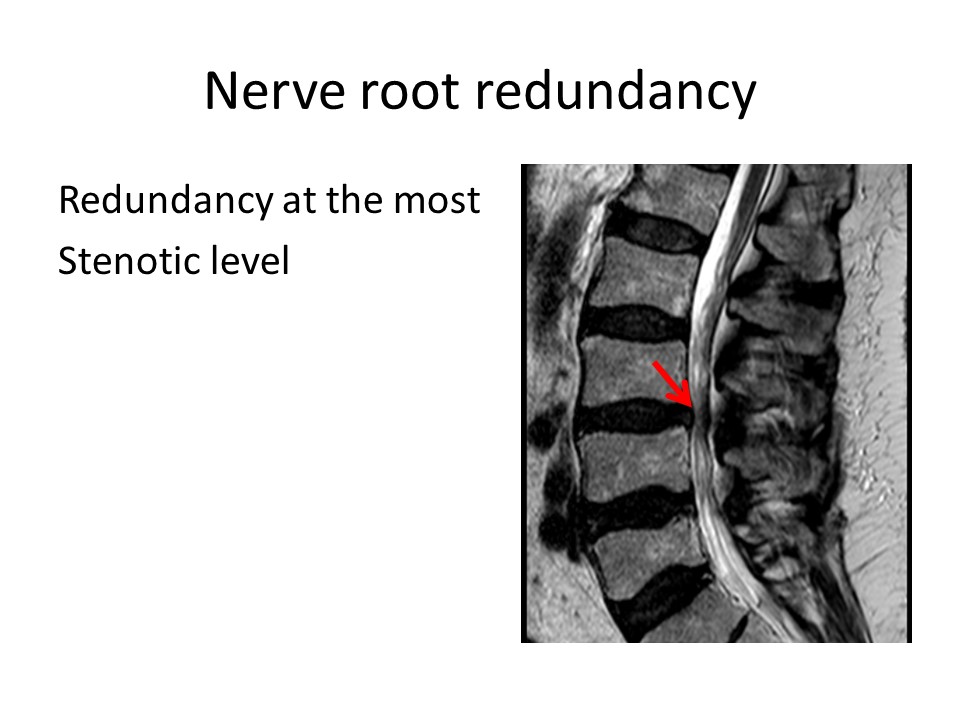


Redundancy of the rootlets of the cauda equina proximal to the stenosis

*Atsushi et al. Clinical significance of the redundant nerve roots of the cauda equina documented on magnetic resonance imaging. Journal of Neurosurgery: Spine SPI. 2007; 7(1):27-32*


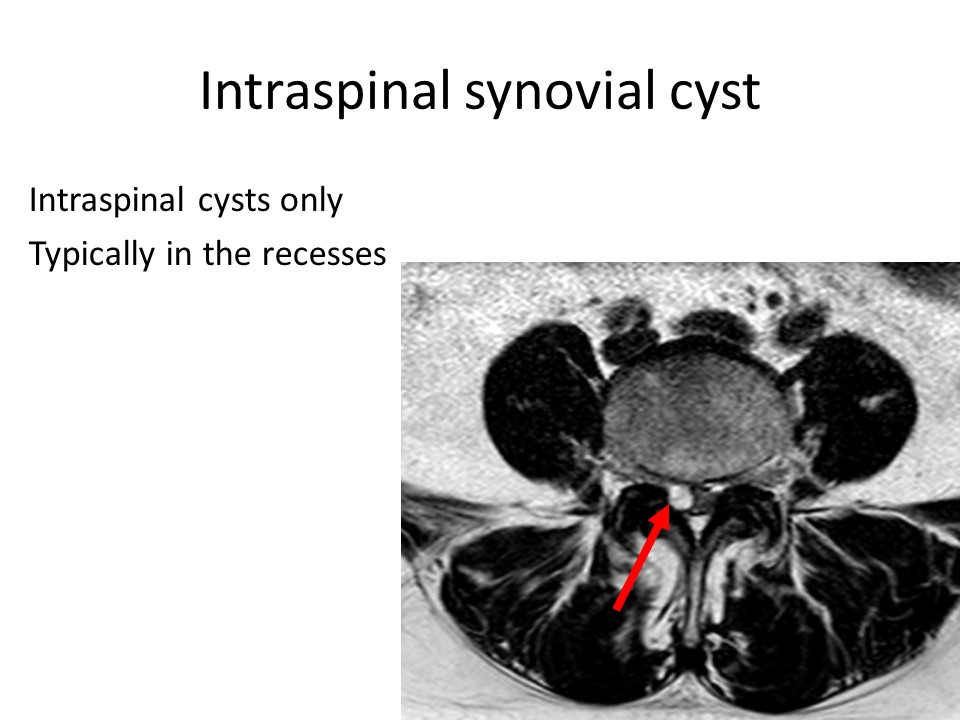


Axial T2-weighted image showing an intraspinal synovial cyst in the right recess

*Boviatsis et al. Spinal synovial cysts: pathogenesis, diagnosis and surgical treatment in a series of seven cases and literature review. Eur Spine J. 2008; 17(6):831-837.*


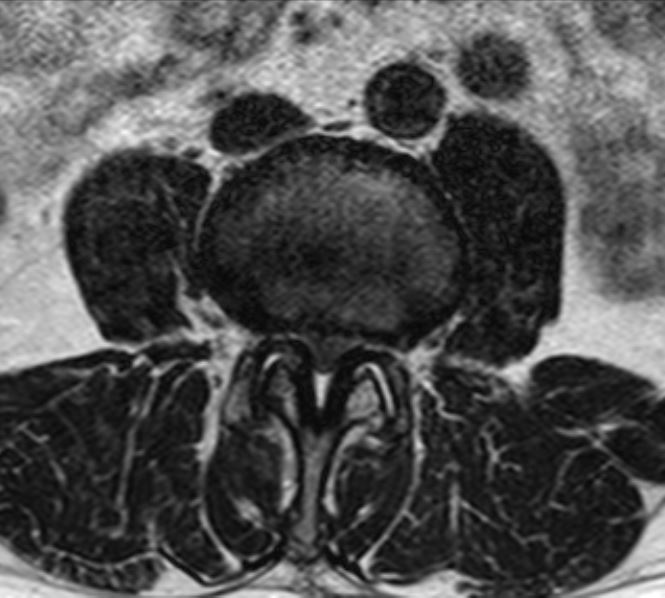


The Dural Sac Cross-sectional Area (DSCA) is measured on axial T2-weighted images at the disc level (stenosis level) by drawing a region of interest on the dural line, excluding the epidural fat.

Measurement of DSCA
